# Human spatial memory is biased towards high-calorie foods: a cross-cultural online experiment

**DOI:** 10.1186/s12966-022-01252-w

**Published:** 2022-02-10

**Authors:** Rachelle de Vries, Sanne Boesveldt, Emely de Vet

**Affiliations:** 1 Sensory Science & Eating Behaviour - Division of Human Nutrition & Health, Wageningen University & Research, P.O. Box 17, Wageningen, 6700 AA The Netherlands; 2 Wageningen University & Research, Consumption & Healthy Lifestyles, Postbus 8130, Wageningen, 6700 EW The Netherlands

**Keywords:** Food spatial memory, Cognitive bias, Cross-cultural analysis, Optimal foraging theory, Eating behaviour, Sociodemographic moderators

## Abstract

**Background:**

Human memory appears to prioritise locations of high-calorie foods, likely as an adaptation for foraging within fluctuating ancestral food environments. Importantly, this “high-calorie bias” in human spatial memory seems to yield consequences for individual eating behaviour in modern food-abundant settings. However, as studies have mainly been conducted in European (Dutch) populations to date, we investigated whether the existence of the cognitive bias can be reasonably generalised across countries that vary on culturally-relevant domains, such as that of the USA and Japan. Furthermore, we investigated whether sociodemographic factors moderate the expression of the high-calorie spatial memory bias in different populations.

**Methods:**

In a cross-cultural online experiment, we measured the food location memory of diverse participants from the USA (*N* = 72; 44.4% Male; 54 ± 15.99 years) and Japan (*N* = 74; 56.8% Male; 50.85 ± 17.32 years), using a validated computer-based spatial memory task with standardised images of high-calorie and low-calorie foods. To directly compare the magnitude of the high-calorie spatial memory bias in a broader cultural scope, we also included data from a previous online experiment that identically tested the food spatial memory of a Dutch sample (*N* = 405; 56.7% Male; 47.57 ± 17.48 years).

**Results:**

In the US sample, individuals more accurately recalled (i.e. had lower pointing errors for) locations of high-calorie foods versus that of low-calorie alternatives (*Mean difference* = -99.23 pixels, 95% CI = [-197.19, -1.28]) – regardless of one’s hedonic preferences, familiarity with foods, and encoding times. Likewise, individuals in the Japanese sample displayed an enhanced memory for locations of high-calorie (savoury-tasting) foods (*Mean difference* = -40.41 pixels, 95% CI = [-76.14, -4.68]), while controlling for the same set of potential confounders. The magnitude of the high-calorie bias in spatial memory was similar across populations (i.e. the USA, Japan, and the Netherlands), as well as across diverse sociodemographic groups within a population.

**Conclusions:**

Our results demonstrate that the high-calorie bias in spatial memory transcends sociocultural boundaries. Since the cognitive bias may negatively impact on our dietary decisions, it would be wise to invest in strategies that intervene on our seemingly universal ability to efficiently locate calorie-rich foods.

**Supplementary Information:**

The online version contains supplementary material available at 10.1186/s12966-022-01252-w.

## Introduction

Essentially all organisms require energy from food to survive and reproduce [1]. Many nutritional ecology models predicting individuals’ eating behaviour thus make the fundamental assumption that natural selection favoured “optimal” foraging strategies that maximize the rate of energy gain [1–3]. Although evolved optimal foraging mechanisms have long been recognised in various animal species – ranging from birds [4–7] to non-human primates (e.g. [8–10]) – the existence of similar foraging-related cognitive adaptations in humans has received less attention in literature [11–13].

In a series of recent studies, we accumulated evidence consistent with the existence of a cognitive mechanism in humans that seems to be geared for the efficient location of fitness-relevant nutritional resources. Namely, we found that human memory shows sensitivity to the caloric quality of a potential food, and more accurately recalls the locations of those higher in energy density (i.e. with a higher amount of energy (kcal) per weight of food) ([14, 15]; see also [12]). The spatial prioritisation of high-calorie foods (e.g. potato chips and chocolate) in memory occurred independently of the nature of the food stimuli (i.e. food images, food odours, or actual food products), an individual’s personal dietary preferences or familiarity with a food, and even one’s conscious effort or explicit instruction to encode food locations (cf. [14]). Importantly, outcomes of earlier investigations indicate that this implicit “high-calorie bias” in human spatial memory may yield consequences for how individuals navigate modern food-abundant environments: An increased expression of the cognitive bias (i.e. a further improved memory for locations of high-calorie versus low-calorie foods) was found to predict a greater reported ease of finding high-calorie foods in a supermarket, more routine visits to high-calorie food retail locations (e.g. fast-food outlets), a stronger habit of purchasing high-calorie snack foods and a subsequently higher (less healthy) BMI [16–18].

The evolutionary focus of the spatial processing bias strongly implies it to be part of our universal (human) cognitive architecture, thus its expression should be reliably observed across cultures [11, 19, 20]. However, as studies have predominantly been conducted in European (Dutch) populations to date, our primary research objective was to investigate whether the existence of the high-calorie bias in spatial memory can be generalised to members of other countries (i.e. USA and Japan), which deviate from one another on domains relevant for food-specific spatial memory. Specifically, “Western” North American and “Eastern” Japanese cultures show dissimilar ways of cognitively processing information, such as in the perception of visual objects and their (spatiotemporal) contexts [21–23], as well as the description of spatial relations between objects [11, 24]. Relatedly, the physical layout of food environments varies between these countries, such as the distribution of spatial cues (e.g. street name signs) and food retailers (e.g. “unhealthy” fast-food outlets versus “healthy” supermarkets). For instance, Japanese cities contain relatively less marked streets compared to US counterparts [24, 25]. On the other hand, in the US there is a clearer prevalence of unhealthy “food deserts” [26], lower market dominance of supermarkets [27], and a higher density of fast-food outlets [28] as compared to Japan and European countries. These environmental differences may impact on the spatial reference strategies (e.g. reliance on landmarks) that individuals habitually adopt, as well as the types of foods that individuals regularly consume [24, 25, 28–30]. Finally, attitudes towards food and the assumed role food plays in daily life are known to vary cross-culturally: European individuals generally associate food most with pleasure, whereas American individuals typically emphasize the health (as opposed to hedonic) utility of food, and Asian (i.e. Japanese and Chinese) individuals tend to view food as either a medium for health or pleasure ([31–33]; see [34] for cultural differences in the “unhealthy food = tasty” intuition).

Given the apparent behavioural and health implications of the high-calorie spatial memory bias, our secondary research objective was to identify potential “at-risk” subgroups within a population that showcase a high expression of the bias. Literature increasingly suggests that dietary patterns and BMI follow a sociodemographic gradient, in that individuals with a lower socioeconomic position (i.e. lower income, education, occupational status, or perceived social standing) generally exhibit poorer diets and a higher body weight [35–37]. The latter is often attributed to the fact that socially disadvantaged individuals tend to be exposed to less healthy physical food environments with an increased availability and accessibility to high-calorie foods [38–40]. A novel (partial) explanation for how these structural differences in local food environments can give rise to dietary disparities between sociodemographic classes could be that the magnitude of the high-calorie spatial memory bias systematically differs between groups. To explicitly assess this, we cross-culturally examined whether sociodemographic factors moderate the expression of the high-calorie spatial memory bias.

Taken together, we hypothesized that:

- *H*_*1A*_*:* The high-calorie spatial memory bias is expressed across different cultures. Specifically, individuals within a country (i.e. USA and Japan) will display a greater overall accuracy in spatial memory for high-calorie foods compared to low-calorie alternatives – regardless of subjective evaluations and familiarity with foods.
- *H*_*1B*_*:* Sociodemographic factors moderate the expression of the high-calorie bias in human food spatial memory within a population.

## Methodology

### Design

The present study had a two-by-two mixed factorial design with *Country* (USA versus Japan) as a between-subjects factor and *Caloric Density* (High versus Low) as a within-subjects factor. In an online experiment, participants had to complete food-specific spatial memory tasks and a series of questionnaires. The hypotheses, experimental design, and statistical analysis plan were preregistered, and are accessible with study data on the Open Science Framework database (Project URL: https://osf.io/ptgda/). For a final exploratory analysis, we included data from a previous online experiment that tested the food spatial memory of a Dutch sample in an identical manner (Project URL: https://osf.io/nv7a9/; 17). The latter was performed to directly compare the magnitude of the high-calorie spatial memory bias in a broader cultural scope (i.e. between American, Asian, and European populations; see [31–33]). This study was approved by the Social Sciences Ethics Committee of Wageningen University.

### Participants

Participants were a diverse sample of healthy adults (above the age of 18) from the USA and Japan, respectively. Individuals were recruited by the ISO-certified *Flycatcher* online research agency (www.flycatcher.eu), using stratified sampling methods (i.e. on background characteristics such as sex and age) in panels of both countries, similar to our former online study [17]. An invite with a link to the online experiment was sent out via email to panellists in both countries who fit the general targeted demographic (i.e. adults from the USA and Japan). An individual was initially screened and excluded from participating in the case of any self-reported illnesses or dietary restrictions, a current or medical history of eating disorders, or (total or partial) colour blindness. A total of 121 panellists responded from the US population, but 43 (35.5%) participants did not fulfil one or more selection criteria (i.e. 20 (16.5%) self-reported poor health or a pre-existing health condition; 5 (4.1%) reported colour blindness; 18 (14.9%) reported food restrictions), 5 (4.1%) dropped-out prior to the food spatial memory task, and 1 participant (0.8%) was removed due to poor response quality. A final sample of 72 individuals (44.4% Male; M_Age_ = 54 (± 15.99) years, Interdecile Range: 32 – 76 years) was thus obtained from the USA. With regards to the Japanese population, 191 panellists initially responded, with 116 (60.7%) individuals excluded at the beginning of the experiment on the basis of our selection criteria (i.e. 104 (54.5%) self-reported poor health or a pre-existing health condition; 1 (0.5%) reported colour blindness; 11 (5.8%) reported food restrictions), and 1 (0.5%) dropped-out prior to the food spatial memory task. Therefore, data from 74 individuals (56.8% Male; M_Age_ = 50.85 (± 17.32) years, Interdecile Range: 29 – 75 years) were collected for the Japanese sample. *A priori* power calculations for our confirmatory analyses (see https://osf.io/ptgda/) were based on earlier investigations with similar within-subjects experimental designs (see [17, 18]), and performed at the smallest possible size of Caloric Density effects (i.e. partial eta squared (ηp^2^) of 0.007) and a power of 0.80. The latter yielded a minimum number of 70 individuals to detect the high-calorie spatial memory bias within a population. Final participant samples of the two countries had comparable sociodemographic distributions (Table 1). For our exploratory multi-country analysis, data pulled from the Dutch sample consisted of 405 individuals that fulfilled the same inclusion and exclusion criteria (56.7% Male; M_Age_ = 47.57 (± 17.48) years, Interdecile Range: 24 – 70 years; for details see [17]). After providing informed consent and completing the online experiment, participants were debriefed and financially compensated.

**Table 1:** Background characteristics of participant samples across cultures

	**USA (*N* = 72)**	**Japan (*N* = 74)**
**Sex (% Male)**	32 (44.4%)	42 (56.8%)
**Age (years)**	54 (± 16)	50.9 (± 17.3)
	Interdecile Range: 32– 76	Interdecile Range: 29– 75
**Ethnicity (%)**	White: 58 (80.6%)	White: 4 (5.4%)
	Black/African/Caribbean: 5 (6.9%)	Asian: 69 (93.2%)
	Asian: 4 (5.6%)	Not Applicable: 1 (1.4%)
	Latino: 2 (2.8%)	
	Other: 1 (1.4%)	
	Not Applicable: 2 (2.8%)	
**Education (%)**	Elementary school: -	Lower secondary school: 6 (8.1%)
	Middle school: -	Upper secondary general or vocational education: 19 (25.7%)
	High school: 36 (50%)	
	Community College/Junior College: 6 (8.3%)	Associate degree junior college: 1 (1.4%) Associate diploma college of technology: 5 (6.8%)
	University undergraduate: 17 (23.6%) University postgraduate: 13 (18.1%)	Diploma professional training college: 2 (2.7%)
		Advanced diploma professional training college: 3 (4.1%) University undergraduate: 35 (47.3%) University postgraduate: 3 (4.1%)
**Income (%)**	Minimum: 8 (11.1%)	Minimum: 6 (8.1%)
	Below the national average: 22 (30.6%)	Below the national average: 25 (33.8%)
	Approximately the national average: 26 (36.1%)	Approximately the national average: 18 (24.3%)
	1 to 2 times the national average: 7 (9.7%)	1 to 2 times the national average: 14 (18.9%)
	2 or more times the national average: 3 (4.2%)	2 or more times the national average: 9 (12.2%)
	Missing: 6 (8.3%)	Missing: 2 (2.7%)
**Occupation (% Employed)**	32 (44.4%)	50 (67.6%)
**Subjective SES**^**a**^	6 (± 2.0)	5.28 (± 2.0)
	Interdecile Range: 3 – 9	Interdecile Range: 2 – 7.5
**BMI**	27.3 (± 5.5)	22.0 (± 3.2)
	Interdecile Range: 21.5 – 33.8	Interdecile Range: 18.3 – 26.7
	Missing: 2 (2.8%)	Missing: 1 (1.4%)
**Healthy eating goals**^**b**^	5.3 (± 1.1)	5.0 (± 1.3)
	Interdecile Range: 4– 7	Interdecile Range: 3.5– 7

^a ^10-point scale (Adler et al*.* 2000) [35]

^b ^7-point scale (de Vries et al. 2020b) [15]

### Research procedure

Prior to testing, participants were provided with a general cover story of the research, which was to investigate “*what people think about foods that are commonly found within the modern food environment”*. Participants first documented their sociodemographic characteristics (e.g. ethnicity, objective SES, and subjective SES) in a preliminary questionnaire. After, they rated their current hunger state and provided ratings on all (randomly-presented) food stimuli (*N* = 24) on the parameters of *Liking*, *Desire to Eat*, and *Familiarity.* While we did not standardise participants’ food intake prior to testing, we did not expect this to systematically influence our hunger results or adjustment for hunger states (cf. previous lab-based studies [15] and [18]; see [17] for similar hunger controls). Individuals then completed the spatial memory task for both high- and low-calorie foods, with a five-minute break between caloric density conditions. Finally, participants reported their height and weight, as well as answered questions on their healthy eating goals. The latter two questionnaires were presented in a counterbalanced manner (within each country), in order to minimise possible order effects on answers. The online test session took approximately 40 min to complete.

### Apparatus and stimuli

#### Food stimuli in spatial memory task

Images of high- and low-calorie foods were taken from the extended *Food Pics* database, which contains standardised pictures of Western, Asian, and Middle Eastern foods [41]. Foods were considered “high-calorie” if they contained at least 225 kcal per 100 g of food weight (e.g. hamburger, ice cream), and “low-calorie” if they contained at most 60 kcal per 100 g of food weight (e.g. tomato, apple) (cf [15].).

A set of 24 (unbranded) food pictures was used as stimuli for the spatial memory task across all participants, with 12 images of both high- and low-calorie items [17, 18]. The final selection of 24 food images was modified differently for each country, based on results of a pilot study involving a separate sample of the target population in each country (*N* = 31 for the US sample and *N* = 29 for the Japanese sample; Table S1). For both countries, an equal number of sweet and savoury foods were included across caloric density categories, to account for potential taste effects on spatial memory performance (see *Food Stimuli* in the Supplemental Material) [15, 18]. Furthermore, high-calorie stimuli showcased a greater energy density (kcal/100 g) as well as total energy content (kcal) relative to low-calorie alternatives, and were correctly perceived as less healthy and to contain more calories (Table S1). On the other hand, high- and low-calorie foods were matched on macronutrient balance (i.e. protein to carbohydrate and fat ratios; [42]), recognisability, and important perceptual characteristics (e.g. colour and complexity) in the final stimuli sets of both populations (Table S1).

#### Spatial memory task

The computer-based spatial memory task was validated in diverse European samples as an instrument to measure food location memory accuracy [16–18]. Participants were first instructed to imagine that an international food market with 24 food stalls was taking place on an (unfamiliar) university campus. They were then shown 12 images of either high- or low-calorie foods, followed by an image of a map of the university campus with all 24 possible stall locations, at a fixed duration of three seconds each. After this initial viewing phase, the location of the stall selling a food item was indicated on the campus map by a green crosshair, and this was consecutively done for all food stimuli within a caloric density condition (*N* = 12). Individuals then rested for two minutes, after of which they had to perform a series of 12 spatial memory trials. On each trial, participants were randomly-presented with one of the previous food images and required to recall (via mouse-click) its correct assigned stall location on the campus map. All 24 possible stall sites were displayed anew each recall round, and participants could select the same stall location more than once, even though (correct) locations did not overlap between foods. Following a five-minute break, the spatial encoding and recall procedure was repeated for the remaining 12 foods of the other caloric density category. Food-location pairs within the campus map, as well as the order in which they were presented, were randomised uniquely for each participant. The order in which individuals completed the spatial memory task between caloric density conditions was counterbalanced within each country. Prior to the actual task with food images, participants first practiced encoding and recalling locations of non-food objects on the campus map, to familiarise themselves with the task paradigm. Importantly, the Japanese version of the spatial memory task (as well as administered questionnaires; *see below*) was translated using official language services, and the online experiment was piloted in a small separate sample (*N* = 3) of native Japanese speakers for clarity and ease of comprehension, following successful internal pre-tests by the research agency.

### Measurements

#### Primary outcome variables

The pointing error, or Euclidian distance (*D*) between correct and recalled stall locations, was averaged across all high- and low-calorie food stimuli to calculate an individual’s spatial memory accuracy for high- and low-calorie foods, respectively [15, 16]. As such, *lower D* scores indicate a *higher* accuracy in food spatial memory. The difference in spatial memory accuracy for high- versus low-calorie foods ((*D*_High Calorie_ – *D*_Low Calorie_) of each individual was taken to represent the high-calorie bias in spatial memory. It follows that *lower* (negative) values denote an *increased* expression of the high-calorie spatial memory bias.

#### Predictor variables

Information on the sociodemographic variables of sex, age, ethnicity, objective SES (i.e. highest education level, annual household income, and occupation), and subjective SES (10-point MacArthur Subjective Social Status Scale; 35) were collected (Table 1). Ethnicity was defined as the ethnic group an individual most strongly identifies with, and was coded into 7 possible categories for both populations: (1) White (2) Black/African/Caribbean (3) Asian (4) Latino (5) Arab (6) Other (7) Not Applicable [43]. Highest education level followed the national education classification system of a country, spanning from (1) elementary school to (6) university postgraduate for the US population, and from (1) lower secondary school to (8) university postgraduate for the Japanese population. Household income comprised of five categories, with (absolute gross) amounts adjusted for each country: (1) minimum, (2) below the national average, (3) approximately the national average, (4) one to two times the national average, and (5) two or more times the national average (Table 1). Occupation was classified into two groups across samples: (1) currently employed and (2) currently unemployed. Subjective SES, or one’s perceived social standing relative to others, was ranked on a 10-step ladder from (1) closer to those who are worst off in one’s home country (i.e. least money, little or no education, and no job or least respected jobs) to (10) closer to those who are best off in one’s home country (i.e. most money, highest education, and most respected jobs).

#### Control measures

We controlled for a number of aspects relevant to overall (food) spatial memory performance, in order to robustly test for a specific mnemonic effect of a food’s caloric content, as well as increase the predictive validity of our statistical models (cf. Section Data analysis). Given that hedonic evaluations and previous exposure to a food were shown to account for additional variation in food spatial memory accuracy [14, 15, 18], participants rated each item on Liking and Desire to Eat on a 100 mm VAS (anchored from “Not At All” to “Very Much”), as well as Familiarity on a five-point scale [44]. Similarly, hunger states were recorded at the onset of testing using a 100 mm VAS (anchored from “Not At All” to “Very Much”), as hunger may directly amplify the attentional and motivational salience of high-calorie stimuli [45, 46].

As final (exploratory) checks, we required individuals to self-report their height (in *cm* or *feet*) and weight (in *kg* or *pounds*), in order to filter out any residual variance in food spatial memory performance attributable to BMI-related differences in (implicit) food attitudes or overall memory function [47–50]. Likewise, we asked participants to answer a Healthy Eating Goals questionnaire with two items (*In my daily life, I strive to eat healthy*; *It is important to me to eat healthy foods*) rated on a seven-point sale anchored from “Strongly Disagree” to “Strongly Agree”, as a measure of the importance they assigned to dietary self-regulation [15, 51]. The time a participant took to encode a food location (in *milliseconds*) during the cognitive task was also recorded, in order to effectively rule out a general learning account of differences in (spatial) memory performance [14, 15, 18].

### Data analysis

Data was analysed with IBM SPSS Statistics 25. Statistical significance was defined as *p* < 0.05. Fisher’s LSD post-hoc tests were conducted following significant main effects of categorical predictors, as well as significant interaction effects. In addition, significant differences were accompanied by the partial eta squared (ηp^2^) effect size measure, which assesses the proportion of variance in an outcome measure that can be explained by a respective predictor – after accounting for effects of other predictors in a model [52].

#### General model fitting procedure

Food spatial memory data was analysed using a linear mixed model (LMM), which represents a flexible and robust technique to model continuous data with correlated errors [53]. Income information was missing from a minority of participants (i.e. 8.3% and 2.7% from USA and Japan samples, respectively). Highly improbable BMI values of less than 13 kg/m^2^ in 2 instances of the US sample – and less than 16 kg/m^2^ in 1 instance of the Japanese sample [54]– were also removed. However, LMM are generally robust to (conditionally) missing covariate data and the validity of estimated parameters in our final reported models was thus likely preserved [55, 56].

The LMM fitting procedure made use of a backward stepwise approach, in evaluating both the existence and moderation of the high-calorie spatial memory bias across countries. After determining the need for both a random intercept and slope within a model (see tests for random effects in [57]), we first established the covariance structure of random effects in the “saturated” LMM of a sample (i.e. Sections Expression of the high‑calorie spatial memory bias across countries ( H_1A_ and H_1B_) and Moderation of caloric density effects by country (exploratory)), using Restricted Maximum Likelihood (REML) ratio tests and the -2 log likelihood (-2LL) test statistic. Next, we simplified fixed effects of the saturated LMM using Maximum Likelihood (ML) ratio tests and the -2LL test statistic. In all cases, the most parsimonious model was selected for and the final LMM was refitted with REML estimations. The finalised LMM of a sample was cross-checked with a forward stepwise modelling process and fulfilled all necessary assumptions.

#### Expression of the high-calorie spatial memory bias across countries (H_1A_ and H_1B_)

To examine whether the high-calorie bias in human spatial memory generalises across cultures (H_1A_), we formulated a random intercept and slope LMM for each country (*N* = 2), with main and interaction effects of *Caloric Density* and *Taste* as fixed factors, *Participant* and *Time* as random factors (covariance structure: Unstructured), *Sex*, *Age*, *Ethnicity*, *Education*, *Household Income*, *Occupation*, *Subjective SES*, *Liking, Desirability*, *Familiarity,* and *Hunger* as covariates, and *Spatial Memory Accuracy* (*D*) as the dependent variable.

To test for (country-specific) sociodemographic moderators of the bias (H_1B_), we included respective interactions between *Caloric Density* and all sociodemographic factors as additional fixed effects in the saturated LMM of each population (outlined above).

#### Moderation of caloric density effects by country (exploratory)

We combined data of the present study with that of a previous online experiment that measured the food spatial memory of individuals from the Netherlands in an identical manner (17), to obtain sufficient power and test whether the magnitude of the high-calorie spatial memory bias differs across populations. We conducted a (random intercept and slope) LMM analysis on the combined dataset of 3 countries (i.e. USA, Japan, and the Netherlands), with main and interaction effects of *Country*, *Caloric Density* and *Taste* as fixed factors, *Participant* and *Time* as random factors (covariance structure: Unstructured), *Sex*, *Age*, *Ethnicity*, *Education*, *Household Income*, *Occupation*, *Subjective SES*, *Liking, Desirability*, *Familiarity,* and *Hunger* as covariates, and log_10_ (y + 1) transformed *Spatial Memory Accuracy* (*D*) as the dependent variable. Since education classification systems differ per country, we standardised the categorisation of Education into 7 possible levels, ranging from (1) primary education to (7) university postgraduate, following the International Standard Classification of Education (ISCED) guidelines [58].

We included individual interactions between *Caloric Density* and sociodemographic factors in the same LMM, to explore whether moderation effects would hold in a broader cultural context.

## Results

### The high-calorie bias in spatial memory was demonstrated across sociodemographic groups in the US sample

Individuals in the US sample exhibited an average food spatial memory accuracy (i.e. average pointing error or *D*) of 316.64 pixels (*SD* = 209.10; Interdecile Range = 14.40 – 589.47).

**Fig. 1:**
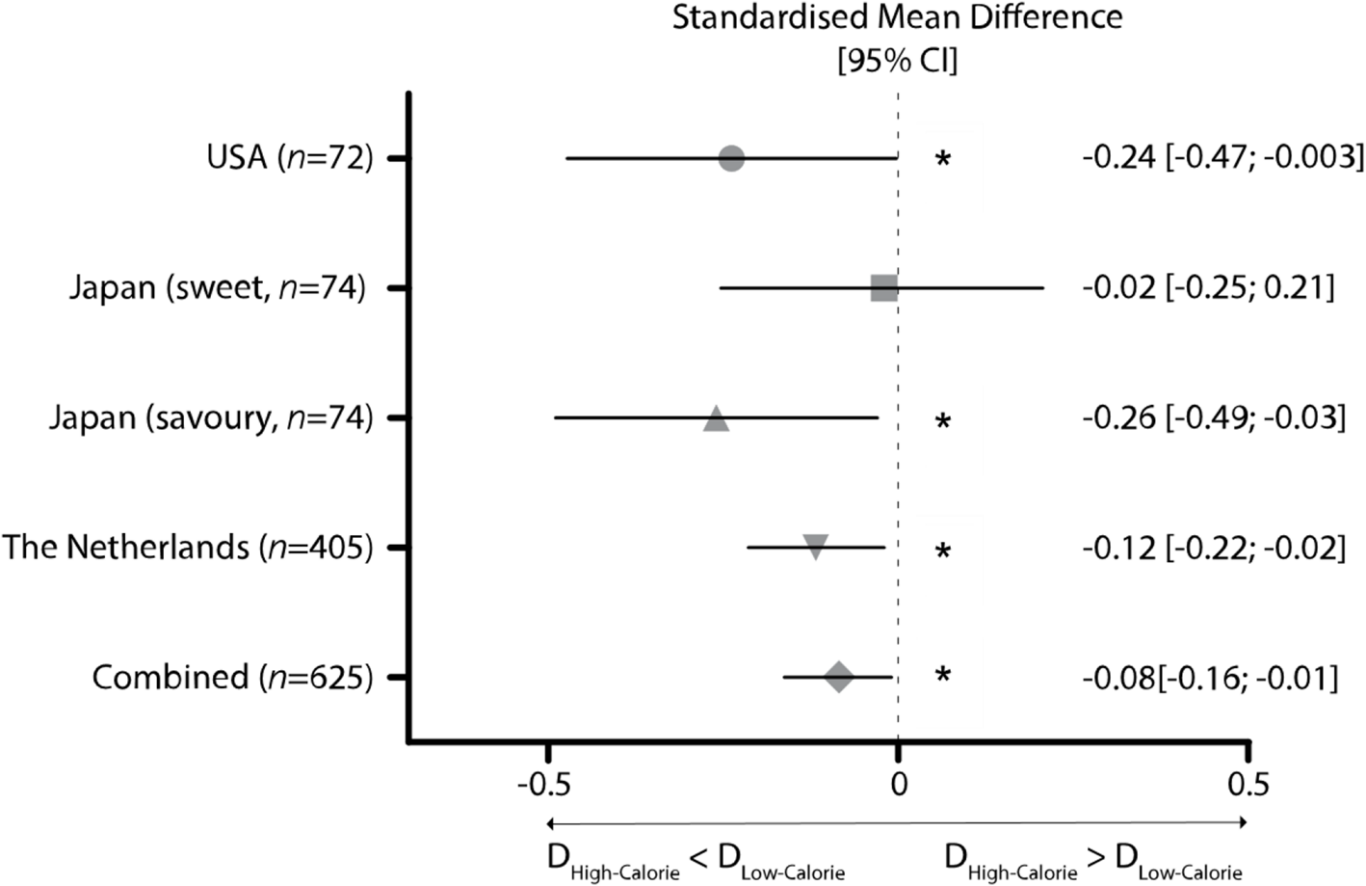
Standardised mean differences in spatial memory performance (*D*) for high-calorie versus low-calorie foods across populations. Lower (negative) values indicate a greater accuracy in spatial memory for high-calorie foods. A significant Caloric Density*Taste interaction was found in the Japanese sample, thus differences are stratified per Taste group. An asterisk denotes a significant expression of the high-calorie spatial memory bias within a population at *p* < 0.05

The Caloric Density of a food was a significant predictor of how accurate its location was later recalled, F(1,70) = 4.08, *p* = 0.047, as individuals demonstrated lower pointing errors on average for locations of high-calorie foods relative to low-calorie alternatives (Table 2 and Fig. 1). The enhanced memory for high-calorie food locations remained significant after adjusting for one’s sociodemographic characteristics, hunger state, food liking, wanting of a food, and familiarity with a food. Further, a food’s caloric content was found to exert a medium sized influence, as it accounted for 6% of the variation in food spatial memory data, after partialling out effects of other predictors,ηp^2^ = 0.06, 90%CI ηp^2^ = [0.0004, 0.16]. Among tested covariates, spatial memory performance was also shown to be influenced to a larger degree by an individual’s Ethnicity, in which Black/African/Caribbean individuals generally displayed larger pointing errors than those from Caucasian, Asian, and Latino ethnic groups (Table 2). Moreover, final checks revealed that pointing errors decreased by 3 pixels for every (second) increase in encoding time (B = -0.003), and increased by 5.12 pixels for every unit rise in BMI (B = 5.12) – with similar sized influences on spatial memory performance as Caloric Density (Table 2). However, none of these additional controls attenuated the effect of – or variation in food spatial memory accounted for by – Caloric Density, F(1,68) = 4.26, *p* = 0.043. The spatial recall of a food did not depend on its Taste quality (i.e. sweet versus savoury; Table 2).

With regards to sociodemographic moderators, an initial trend was found for the interaction between Caloric Density and Education, F(2,67) = 2.58, *p* = 0.083, in which a higher education level tended to improve spatial memory performance for low-calorie foods only (Table 2). However, this trend was eliminated upon controlling for individuals’ BMI, F(2,63) = 2.12, *p* = 0.128. None of the remaining interactions proved to be significant, indicating that the high-calorie bias in spatial memory was equally expressed across sociodemographic groups in the US sample.

### The high-calorie bias in spatial memory was specific to savoury-tasting foods in the Japanese sample

Individuals in the Japanese sample displayed an average food spatial memory accuracy of 322.05 pixels (*SD* = 236.54; Interdecile Range = 9.84 – 626.48), which mirrored the distribution of pointing errors in the US sample.

After adjusting for sociodemographic variables, hunger, and respective food ratings, a significant but small interaction effect between Caloric Density and Taste on spatial memory performance was observed, F(1,1584) = 4.06, *p* = 0.044 (Table 2). Post-hoc (LSD) comparisons revealed that although individuals showcased lower pointing errors for high- versus low-calorie foods across Taste groups, the difference only reached significance for savoury-tasting items (Table 2 and Fig. 1). In addition, food spatial memory improved to a comparable degree with a higher rated Desirability of a food, and females generally performed better than males (Table 2). On the other hand, neither BMI, reported nutritional intentions, nor encoding time predicted the accuracy of recalling food locations, as shown in final exploratory checks (all *p* > 0.05).

Finally, a significant moderate interaction between Caloric Density and the sociodemographic variable of Occupation was observed, F(2,69) = 3.24, *p* = 0.045 (Table 2), in which employed individuals displayed lower pointing errors for low-calorie foods relative to unemployed individuals. None of the remaining interactions proved to be significant, indicating that locations of high-calorie foods were uniformly prioritised (over that of low-calorie foods) across Japanese sociodemographic groups.

### The magnitude of the high-calorie spatial memory bias was similar across US, Japanese, and Dutch populations

In a combined exploratory analysis of the food spatial memory of individuals from the USA, Japan, and the Netherlands, a significant medium-sized main effect of Country was found, F(2,548) = 25.08, *p* < 0.001, as Dutch participants displayed overall lower pointing errors than both US and Japanese samples (Table 2). Spatial memory performance did not differ between US and Japanese participants, *p* = 0.611.

Controlling for general effects of Country, Caloric Density and Taste were shown to systematically predict food spatial memory accuracy to a small degree, owing to 3.11% lower pointing errors for high-calorie foods, F(1,552) = 4.44, *p* = 0.036, and 2.69% lower pointing errors for savoury-tasting foods, F(1,12,177) = 6.89, *p* = 0.009, respectively (Table 2 and Fig. 1). Importantly however, the interaction between Country and Caloric Density was not significant, *p* = 0.349, suggesting that the enhanced memory for high-calorie food locations was equal in magnitude across tested populations. Of the remaining covariates, a higher Education level and a higher rated Desirability of a food was associated with better spatial memory performance, whereas a male Sex and older Age predicted larger pointing errors, all with comparable effect sizes to Caloric Density (Table 2). Final checks revealed a further small influence of task (encoding) times (Table 2), but this did not mitigate any of the aforementioned effects.

**Table 2:** Finalised linear mixed models (LMM) of food spatial memory performance across cultures

**USA**	**B**^**a**^			**95% CI**		***p***	**ηp**^**2 b**^			**90%CI ηp**^**2**^
**Model 1: Best-fitting LMM**^**c**^										
Intercept	442.98			292.27 – 593.69		< .001*	-			-
Caloric Density (High – Low)	-99.23			-197.19—-1.28		.047*	0.06			0.0004 – 0.16
Taste (Sweet – Savoury)	-5.32			-22.58 – 11.95		.546	-			-
Ethnicity	-			-		.041*	0.16			0.02 – 0.63
Caucasian—Black	-135.70			-222.10—-49.30		.003*	-			-
Black—Asian	191.45			66.31 – 316.59		.003*	-			-
Black—Latino	168.86			13.50 – 324.21		.034*	-			-
Caloric Density*Education	-			-		.083	-			-
Low*Education	-21.18			-43.16 – 0.80		.059	-			-
**Model 2: Best-fitting LMM with exploratory covariates (i.e. BMI, Healthy Eating Goals, and Encoding Time)**^**c**^										
Intercept	244.44			-10.97 – 499.86		.103	-			-
Caloric Density (High – Low)	-102.22			-201.09—-3.33		.043*	0.06			0.001 – 0.17
Taste (Sweet – Savoury)	-4.52			-22.01 – 12.96		.612	-			-
Ethnicity	-			-		.047*	0.17			0.007 – 0.65
Caucasian—Black	-116.19			-197.69—-34.69		.006*	-			-
Black—Asian	166.22			49.04 – 283.41		.006*	-			-
Black—Latino	196.05			49.71 – 342.39		.010*	-			-
Caloric Density*Education	-			-		.128	-			-
BMI	5.12			1.02 – 9.21		.015*	0.09			0.01 – 0.23
Healthy Eating Goals	2.80			-17.08 – 22.68		.779	-			-
Encoding Time	-0.003			-0.01—-0.001		.001*	0.02			0.01 – 0.05
**Japan**										
**Model 1: Best-fitting LMM (with and without exploratory covariates BMI, Healthy Eating Goals, and Encoding Time)**^**c**^										
Intercept	387.73			314.22 – 461.23		< .001*	-			-
Caloric Density (High – Low)	-69.85			-124.53—-15.17		.161	-			-
Taste (Sweet – Savoury)	-7.89			-33.22 – 17.43		.251	-			-
Desirability	-0.59			-1.01—-0.17		.006*	0.05			0.001 – 0.01
Sex (Males – Females)	70.01			3.28 – 136.75		.040*	0.06			0.002 – 0.17
Caloric Density*Taste	-			-		.044*	0.003			0.0001 – 0.01
High-Savoury – Low-Savoury	-40.41			-76.14—-4.68		.025*	-			-
Caloric Density*Occupation	-			-		.045*	0.09			0.002 – 0.32
Low-Employed – Low-Unemployed	-90.93			-168.89—-12.96		.023*	-			-
**Combined (USA + Japan + Netherlands)**										
**Model 1: Best-fitting LMM **^**d**^										
Intercept	1.83			1.65 – 2.00		< .001*	-			-
Country	-			-		< .001*	0.08			0.09 – 0.22
USA – Netherlands	0.29			0.19 – 0.39		< .001*	-			-
Japan—Netherlands	0.25			0.16 – 0.35		< .001*	-			-
Caloric Density (High – Low)	-0.03			-0.06—-0.002		.036*	0.01			0.0003 – 0.02
Taste (Sweet – Savoury)	0.03			0.01 – 0.05		.009*	0.001			0.0001 – 0.001
Sex (Males – Females)	0.09			0.03 – 0.16		.007*	0.01			0.002 – 0.03
Age	0.007			0.005 – 0.01		< .001*	0.08			0.05 – 0.12
Education	-0.04			-0.07—-0.21		< .001*	0.03			0.01 – 0.05
Desirability	-0.0005			-0.001—-0.0001		.015*	0.001			0.0001 – 0.001
**Model 2: Best-fitting LMM with exploratory covariates (i.e. BMI and Task/Encoding Time) **^**d**^										
Intercept	1.68			1.42 – 1.94		< .001*	-			-
Country	-			-		< .001*	0.09			0.1 – 0.23
USA – Netherlands		0.29	0.19 – 0.39		< .001*			-	-	
Japan—Netherlands		0.29	0.19 – 0.39		< .001*			-	-	
Caloric Density (High – Low)	-0.03			-0.06—-0.002		.037*	0.01			0.0002 – 0.02
Taste (Sweet – Savoury)	0.03			0.007 – 0.05		.009*	0.001			0.0001 – 0.002
Sex (Males – Females)	0.09			0.02 – 0.15		.012*	0.01			0.001 – 0.03
Age	0.007			0.005 – 0.01		< .001*	0.08			0.04 – 0.1
Education	-0.04			-0.06—-0.02		.001*	0.02			0.01 – 0.05
Desirability	-0.0005			-0.001—-0.0001		.015*	0.001			0.0001 – 0.001
BMI	0.005			-0.002 – 0.01		.148	-			-
Task (Encoding) Time	-3.7 × 10^–6^			-5.9—-1.5 × 10^–6^		.001*	0.001			0.0002 – 0.002

^*^Significant at α = 0.05

^a^The change in food spatial memory accuracy (*D; pixels*) associated with a one unit change in the predictor, with other model predictors held constant. Categorical predictors with more than 2 groups (e.g. Ethnicity), as well as interaction effects, are represented by multiple B coefficients – one for each significant group difference as revealed in Fisher’s LSD post-hoc tests

^b^The proportion of variance in food spatial memory that is explained by the predictor, after accounting for effects of other model predictors

^c^A linear mixed model with food spatial memory accuracy (*D; pixels*) as the dependent variable

^d^A linear mixed model with log_10_ (y + 1) transformed food spatial memory accuracy (*D; pixels*) as the dependent variable

## Discussion

The present study reports on a cross-cultural online experiment to investigate the widespread existence of a bias in human spatial cognition for high-calorie foods. We found that individuals native to cultures varying on relevant cognitive characteristics, built food environments, and food attitudes were effectively identical in their food relocation performance: Locations of resources with a higher caloric density were more accurately recalled than that of low-calorie alternatives to a similar degree across countries, regardless of individuals’ hedonic preferences, familiarity with foods, or deliberate effort to encode food locations. In addition, the high-calorie bias in spatial memory was uniformly expressed by diverse sociodemographic groups within a population.

Our investigation is the first to demonstrate that the high-calorie bias in spatial memory transcends cultural boundaries, which empirically strengthens the idea that the mechanism represents a part of our universal (human) cognitive architecture [19, 20, 59]. Though the spatial memory advantage of high-calorie foods was small in magnitude, a food’s caloric density consistently accounted for a comparable amount of variation in pointing errors as general (reward-based) learning mechanisms (e.g. food desirability) across countries. We also adjusted for food encoding times, which can proxy for possible differences in attentional processing between items. Indeed, the unique memory-enhancing quality of a food’s caloric content has been reliably observed in more real-world settings. For instance, a higher caloric density independently predicted a greater odds of correctly recalling a food’s (pillar) location within a room [14], as well as a greater accuracy in pointing to remembered food stall locations within an outdoor farmer’s market [12]. Noteworthy is that while sex differences in spatial cognition are widely documented in literature (e.g. [60]), another replicated result throughout studies is that the bias’ expression is not moderated by sex [12, 14, 17]. Although evolutionary pressures would have likely selected for different spatial skills between ancestral male “hunters” and female “gatherers” [61], an even expression of the bias between sexes makes theoretical sense as the adaptive problem of optimal (energy-efficient) foraging would have been equally relevant for both [62]. Taken together, our results underscore how caloric content serves as an ecologically valid predictor of human food spatial memory.

Importantly, previous research also established that a small one-unit (pixel) improvement in the relative memory accuracy for high-calorie food locations was associated with a subtle but significant change in (long-term) eating behaviour (e.g. 0.001 – 0.01 increase in BMI (kg/m^2^), an anthropometric marker of dietary intake and excess fat mass; [63, 64]), after controlling for more explicit determinants such as an individual’s reported healthy eating intentions [16–18]. Our current findings imply that (individual-level) interventions aiming to reduce the *overall* expression of the cognitive bias to promote healthier dietary regulation would benefit from targeting other predictors of food spatial memory that are more likely under one’s voluntary control (e.g. encoding time). For example, packaging high-calorie products in less vibrant “cool” colours (e.g. blue) [65], or placing them in low traffic areas within food retail outlets, may lower their attentional salience and impair later memory for their corresponding locations. The same principle may be applicable for minimising social dietary inequalities: Training a higher desirability for healthier low-calorie items (e.g. fruits and vegetables) – which is especially compromised in socially disadvantaged groups [66] – can help to offset the underlying spatial recall advantage of energy-dense foods experienced across sociodemographic strata. Indeed, we found some support for such sociodemographic discrepancies in food motivations in our Japanese sample, as unemployed individuals displayed poorer spatial memory performance for specifically low-calorie foods, relative to employed individuals. From a public health perspective, however, a more parsimonious and perhaps effective approach to support healthy eating behaviour on both individual and group levels would be to limit the availability of high-calorie products, as well as high-calorie food locations (e.g. fast food outlets), in the immediate environment. Such structural changes to the physical food environment would help to steer food choice towards healthier alternatives, irrespective of individuals’ responsiveness to proposed cognitive interventions [30, 67].

A major methodological strength of this study is that it made use of a previously unexplored tool (i.e. cross-cultural comparisons) to eliminate competing macro-level explanations for the high-calorie bias in spatial memory (e.g. culture-specific attitudes towards high-calorie foods) [19, 20, 59]. Furthermore, we managed to recruit a diverse pool of participants from each culture – including those that are typically hard-to-reach (e.g. low SES groups) – and closely-matched high-calorie and low-calorie food stimuli across countries. That said, we did encounter some limitations. Firstly, our US and Japanese samples were not fully representative of respective adult populations at a national scale, and were smaller in size compared to the Dutch sample. This possibly resulted in less precise estimates (i.e. slightly higher standard errors and wider confidence intervals) of the spatial memory performance of US and Japanese populations. Nevertheless, standard errors of obtained estimates were still acceptable and samples had similar sociodemographic distributions – allowing for a fair and sufficiently accurate comparison of caloric density effects between cultures, which was our primary research interest. Moreover, one could argue that our resulting food images were still more “westernised” in nature, despite efforts to tailor them cross-culturally. The latter could have contributed to the initial Caloric Density-Taste interaction observed in our Japan analysis, as more contextually-appropriate items were available for our savoury-tasting stimuli (e.g. ramen). We believe this speaks to a greater need for food image databases (e.g. [41, 68]) to amplify existing efforts to diversify their collection of standardised pictures and increase cross-cultural applicability. Finally, given that a priori power calculations were based on our primary objective of detecting main Caloric Density effects within distinct samples, it is likely that tests for certain interaction effects (i.e. Caloric Density*Ethnicity) were underpowered. Potential differences between ethnicities should be re-examined in particular, as ethnic groups were unevenly represented within more varied populations (i.e. the USA).

## Conclusions

In closing, diverse sociodemographic groups from three distinct cultures were shown to display an identical food spatial memory “signature”: individuals automatically prioritised in memory the locations of foods with a higher caloric content. Since the high-calorie bias in spatial memory may negatively impact on our dietary decisions, it would be wise to invest in strategies that intervene on our seemingly universal ability to efficiently locate calorie-rich foods.

## Supplementary Information

**Additional file 1: Table S1.** Characteristics of high- and low-calorie food stimuli used in the spatial memory task across cultures. **Food Stimuli. **High- and low-calorie food items used in the spatial memory task of each country.

## Data Availability

The datasets generated and/or analysed during the current study are available in the Open Science Framework repository, https://osf.io/ptgda/.
