# Supplementary material for: Human spatial memory is biased towards high-calorie foods: a cross-cultural online experiment
Source: Int J Behav Nutr Phys Act. 2022 Feb 10;19:14. doi: 10.1186/s12966-022-01252-w (PMC8832830; doi:10.1186/s12966-022-01252-w)
Supplement: Supplementary file 1 — Additional file 1: Table S1. Characteristics of high- and low-calorie food stimuli used in the spatial memory task across cultures. Food Stimuli. High- and low-calorie food items used in the spatial memory task of each country. [file 12966_2022_1252_MOESM1_ESM.docx]

**Supplemental Material for:**

Human spatial memory is biased towards high-calorie foods: A cross-cultural online experiment

**This file includes:**

Table S1

Food Stimuli

**Table S1.**

Characteristics of high- and low-calorie food stimuli used in the spatial memory task across cultures.

| **Spatial Memory Task - USA** | **Parameter (*unit*)** | **High-calorie**  **M (SD)** | **Low-calorie**  **M (SD)** | ***p*** |
| --- | --- | --- | --- | --- |
| **Nutritional Content ^a^** | Energy density (*kcal/100g*) | 381.3 (111.7) | 30.8 (16.1) | <.001 |
|  | Total energy displayed (*kcal*) | 677.9 (797.8) | 115.4 (112.1) | <.001 |
|  | Protein to Carbohydrate and Fat Ratio | 0.2 (0.2) | 0.4 (0.4) | .514 |
| **Subjective Ratings ^b^** | Perceived calories (*mm*) | 85.9 (19.6) | 29.3 (23.3) | <.001 |
|  | Perceived healthiness (*mm*) | 21.3 (23.6) | 78 (24.2) | <.001 |
|  | Recognisability (*%*) | 83.6 (6.7) | 81.16 (6.7) | .381 |
| **Image Characteristics ^a^** | Colour (Red; *pixels*) | 0.5 (0.03) | 0.5 (0.1) | .796 |
|  | Colour (Green; *pixels*) | 0.3 (0.03) | 0.4 (0.1) | .211 |
|  | Colour (Blue; *pixels*) | 0.2 (0.03) | 0.2 (0.1) | .159 |
|  | Size *(pixels)* | 0.3 (0.1) | 0.3 (0.1) | .861 |
|  | Brightness *(luminance)* | 33.3 (9.2) | 31.5 (8.3) | .620 |
|  | Contrast *(luminance)* | 48.2 (9.2) | 45.2 (13.2) | .518 |
|  | Complexity *(pixels)* | 0.1 (0.02) | 0.1 (0.03) | .602 |
|  | Normalised Complexity *(pixels)* | 0.3 (0.1) | 0.3 (0.1) | .985 |

**^a^** Data available from the *Food Pics* database (Blechert *et al*., 2019).

**^b^** Pilot results from a separate sample of the target population (N = 31; 45.2% Male; M_Age_ = 51.5 (± 16.4) years, Range: 23 – 85 years)

| **Spatial Memory Task - Japan** | **Parameter (*unit*)** | **High-calorie**  **M (SD)** | **Low-calorie**  **M (SD)** | ***p*** |
| --- | --- | --- | --- | --- |
| **Nutritional Content ^a^** | Energy density (*kcal/100g*) | 378 (109.1) | 32 (16.3) | <.001 |
|  | Total energy displayed (*kcal*) | 692.1 (788.5) | 119.9 (109.1) | <.001 |
|  | Protein to Carbohydrate and Fat Ratio | 0.2 (0.2) | 0.3 (0.4) | .755 |
| **Subjective Ratings ^b^** | Perceived calories (*mm*) | 80.8 (17) | 41.8 (25.5) | <.001 |
|  | Perceived healthiness (*mm*) | 36.4 (26.9) | 73.9 (19.9) | <.001 |
|  | Recognisability (*%*) | 91.1 (5.2) | 91.7 (7.4) | .823 |
| **Image Characteristics ^a^** | Colour (Red; *pixels*) | 0.5 (0.03) | 0.5 (01) | .729 |
|  | Colour (Green; *pixels*) | 0.3 (0.03) | 0.4 (0.1) | .223 |
|  | Colour (Blue; *pixels*) | 0.2 (0.03) | 0.2 (0.1) | .300 |
|  | Size *(pixels)* | 0.3 (0.1) | 0.3 (0.1) | .426 |
|  | Brightness *(luminance)* | 37.1 (13) | 30.6 (9.1) | .169 |
|  | Contrast *(luminance)* | 49.5 (8.2) | 46.1 (13.4) | .463 |
|  | Complexity *(pixels)* | 0.1 (0.03) | 0.1 (0.03) | .114 |
|  | Normalised Complexity *(pixels)* | 0.3 (0.1) | 0.3 (0.1) | .927 |

**^a^** Data available from the extended *Food Pics* database (Blechert *et al*., 2019).

**^b^** Pilot results from a separate sample of the target population (N = 29; 48.3% Male; M_Age_ = 48.6 (± 16.5) years, Range: 19 – 80 years)

**Food Stimuli**

High- and low-calorie food items used in the spatial memory task of each country.

| **USA** | | ***Food Pics* catalogue number ^a^** | **Example Foods ^a^** |
| --- | --- | --- | --- |
| **High-calorie** | **Sweet** | 4  16  103  116  134  286 | Chocolate chip cookie; Pancakes; Chocolate muffin |
|  | **Savoury** | 2  27  53  60  104  517 | Hamburger; Fries; Hotdog |
| **Low-calorie** | **Sweet** | 199  389  392  413  453  466 | Watermelon; Apple; Kiwis |
|  | **Savoury** | 233  250  251  260  364  442 | Tomatoes; Green beans; Bell pepper |

**^a^** From the extended *Food Pics* database (Blechert *et al*., 2019).

| **Japan** | | ***Food Pics* catalogue number ^a^** | **Example Foods ^a^** |
| --- | --- | --- | --- |
| **High-calorie** | **Sweet** | 4  16  103  116  134  286 | Cake; Ice cream; Chocolate bar |
|  | **Savoury** | 2  27  53  60  517  619 | Potato chips; Fries; Ramen noodles |
| **Low-calorie** | **Sweet** | 199  389  392  413  453  466 | Cantaloupe; Mandarins; Peach |
|  | **Savoury** | 233  250  251  260  333  442 | Broccoli; Cabbage; Spring onions |

**^a^** From the extended *Food Pics* database (Blechert *et al*., 2019).
